# Nest density drives productivity in chestnut-collared longspurs: Implications for grassland bird conservation

**DOI:** 10.1371/journal.pone.0256346

**Published:** 2021-08-24

**Authors:** John P. Pulliam, Scott Somershoe, Marisa Sather, Lance B. McNew

**Affiliations:** 1 Department of Animal & Range Sciences, Montana State University, Bozeman, Montana, United States of America; 2 Division of Habitat Conservation, Land Bird Coordinator U.S. Fish and Wildlife Service Migratory Birds Program, Lakewood, Colorado, United States of America; 3 Wildlife Biologist, Partners for Fish and Wildlife Program, U.S. Fish and Wildlife Service, Glasgow, Montana, United States of America; 4 Department of Animal & Range Sciences, Montana State University, Bozeman, Montana, United States of America; The Clifton Institute, UNITED STATES

## Abstract

Grassland birds are declining faster than any other avian guild in North America and are increasingly a focus of conservation concern. Adaptive, outcome-based management of rangelands could do much to mitigate declines. However, this approach relies on quantitative, generalizable habitat targets that have been difficult to extrapolate from the literature. Past work relies heavily on individual versus population response, and direct response to management (e.g. grazing) versus response to outcomes. We compared individual and population-level responses to vegetation conditions across scales to identify quantitative targets of habitat quality for an imperiled grassland songbird, the chestnut-collared longspur (*Calcarius ornatus*) in northern Montana, USA during 2017–2018. We estimated nest density and nest survival within 9-ha survey plots using open N-mixture and nest survival models, respectively, and evaluated relationships with plot- and nest-site vegetation conditions. Plot-scale conditions influenced nest density, whereas nest survival was unaffected by any measured condition. Nest-site and plot-scale vegetation measurements were only weakly correlated, suggesting that management targets based on nest sites only would be incomplete. While nest survival is often assumed to be the key driver of bird productivity, our results suggest that nest density and plot-scale conditions are more important for productivity of longspurs at the core of the breeding distribution. Habitat outcomes for grassland birds should incorporate nest density and average conditions at scale(s) relevant to management (e.g. paddock or pasture).

## Introduction

The decline of grassland bird populations in North America during the past 40 years [1,2] has increased interest in managing habitats for these species. However, practical guidelines for rangeland management to benefit birds, such as grazing or burning regimes, has been difficult to generalize because species response is highly dependent on local context. Somershoe et al. [3] reviewed the effects of grazing management on four priority grassland bird species of the northern Great Plains. They found that each species was shown to respond positively, negatively or not at all to grazing, depending on local context. For example, season-long grazing was associated with increased abundance of chestnut-collared longspurs (*Calcarius ornatus*) in Alberta [4,5], decreased abundance in North Dakota [6] and had no effect on birds in Montana [7]. Lipsey and Naugle [8] showed that response to grazing across several grassland species was highly dependent on past precipitation and soil productivity.

Due to the complexities of weather, soils, plant communities, topography and historical land use, there is increasing support for a strategy of “adaptive management for outcomes” in rangelands [9]. This approach leverages the skills and knowledge of local land managers to produce quantifiable outcomes or targets which are then monitored adaptively. The methods for achieving a given outcome might differ substantially in different contexts, but this becomes less important because the outcome itself is the focus. Indeed, the U.S. Bureau of Land Management (BLM), one of the largest rangeland managers in North America, is moving towards “outcome-based grazing” in several regions, with outcomes linked to reducing invasive annual grass encroachment and fire risk [10].

The outcome-based approach holds great promise to unify rangeland management recommendations for grassland birds in spite of contextual variability across the breeding range. However, the task of quantifying specific outcomes associated with high habitat quality for these species becomes critical. Habitat quality is highly scale-dependent and can differ depending on whether individual or population-scale responses are considered [11]. For example, an area with a few rich concentrations of resources can be highly beneficial to certain individuals, whereas an area with abundant, lower-quality resources might support a larger, more persistent population [11]. One limitation of grassland bird-habitat research to date is that the scale of variables quantified, in general, has not matched the scale of population response to management. Research has primarily focused on individual response to local-scale conditions (i.e., at a nest) whereas management affects large areas and is more likely to elicit population-level response (e.g., at a pasture or ranch scale). Here, we compare individual and population-level responses to vegetation conditions across scales to identify quantifiable components of habitat quality (i.e., management outcomes) for an imperiled grassland songbird in Montana, the chestnut-collared longspur (hereafter “longspur”).

Ecological theory emphasizes habitat quality for individuals over groups or populations because natural selection acts on the individual [11]. By contrast, populations are often the focus of conservation management. For songbirds, habitat quality has typically been inferred from nest survival under the assumption that conditions associated with successful nests are optimal for population productivity [12,13]. However, if factors influencing nest placement or initiation decisions differ from those affecting nest survival, then management recommendations based on survival alone may be inadequate or even inappropriate. Conditions promoting high nest densities could have greater influence on population-level productivity than conditions associated with individual nest survival [14].

Scale mismatch between independent (habitat) variables and management actions represents another limitation to the development of effective management guidelines for grassland birds. Previous research has typically measured vegetation conditions at nest-sites and compared these to nearby random points or transects [15–17]. This body of work has identified important conditions associated with nest survival. However, extrapolation of these findings to the scale of management actions (e.g., paddock) relies on the assumption that conditions at locations used by wildlife, such as nest-sites, are closely correlated to conditions at the broader scale. If this assumption is violated, outcomes based on nest site vegetation parameters have limited utility to land managers.

We used longspurs as a model to separate and evaluate these components of population productivity in an area of northern Montana representing the core of their extant distribution. Chestnut-collared longspurs are of particular interest for conservation and management due to their severe rates of population decline, estimated at 4.2% annually with an overall decline of 87.3% since the 1960’s [1,3]. Declines are variously attributed to conversion, fragmentation, and degradation of grasslands and the resulting loss of high-quality nesting habitat [3,16], but mechanisms of population performance are generally unknown. Our objective was to define management outcomes for this species that would be most closely linked to population productivity. Specifically, we evaluate: 1) the relationship between nest density and nest survival, 2) the relationship between nest-site and plot-scale vegetation conditions, 3) the effect of plot-scale conditions on nest density, and 4) the effects of nest-site and plot-scale conditions on nest survival and overall population productivity.

## Materials and methods

### Study area

Our study area was located in northern Phillips County, Montana, ranging from 24 km south of Malta, MT to eight km south of the Canadian border (see S1 Fig in supplementary materials). This region contains one of the largest tracts of intact native mixed-grass prairie remaining in the U.S. [18]. Range-wide models of breeding longspurs predict that Phillips County contains some of the highest relative densities in North America [19]. The region is dominated by gently rolling grassy hills. The climate is semi-arid with short, hot summers and long, cold winters, average daily temperatures range from -14° C in January to 28° C in July [18]. Average annual precipitation typically comes as rain in May–July [18], and ranges from 193–493 mm per year (1981–2015; [20]). The dominant grasses are western wheatgrass (*Pascopyrum smithii*), needle-and-thread (*Hesperostipa comata*), prairie junegrass (*Koeleria macrantha*), green needlegrass (*Stipa viridula)*, and Sandberg bluegrass (*Poa secunda*) [21]. Almost 75% of the region is managed for livestock grazing; continuous season-long grazing and low to moderate stocking rates (0.3–1.2 AUM ha^-1^) are the norm [19]. The exotic grass species observed in the area included crested wheatgrass (*Agropyron cristatum*), smooth brome (*Bromus inermis*), and Kentucky bluegrass (*Poa pratensis*).

### Site selection

To select study plots, we used a stratified random sampling approach to minimize the effect of unmeasured sources of variation, including soil characteristics, expected annual precipitation, and distance to roads [8,22]. Soil survey data were downloaded from the Natural Resource Conservation Service (NRCS) web soil survey dataset [23] and used to select silty ecological sites with 250–330 mm of average precipitation and at least 1,120 kg ha^-1^ of vegetation productivity in a normal year. These site conditions have been previously identified to support longspurs [8]. We used ArcMap 10.4.1 [24] to randomly generate 152 potential survey plots measuring 300 × 300 m. We selected plots that were completely within one land ownership type and were > 100 m from a road [22]. Only 28.4% of the study area met suitability criteria, resulting in plots that were spatially clustered. On average, plots were 855 m from their nearest neighbors; 90% plots were >100 m from their nearest neighbor (S1 Fig in Supplementary Materials).

To ensure variability in management and vegetation conditions, we selected sites occurring on private and public lands known to have diverse management histories. Ownership included BLM, a U.S. Fish and Wildlife Service National Wildlife Refuge, Montana state trust, and private lands. Because of our focus on management outcomes rather than treatments, we did not collect site-specific information on stocking rates or grazing regimes. Most plots (88%) were grazed by livestock at stocking rates averaging 0.3 AUM ha^-1^ for those occurring on BLM lands (range: 0.04–0.5 AUM ha^-1^; BLM Annual Operating Instructions) and 0.5–1 AUM ha^-1^on state trust lands (M. Sather, person. commun). The predominant grazing system was rotational grazing where cattle were stocked for periods of about a month per pasture between April–November each year. Twelve of 100 plots occurred on ungrazed lands managed by the U.S. Fish and Wildlife Service.

We inspected all plots in 2017 prior to sampling to ensure they did not contain interior fences, roads, or excessive shrub cover which would negatively impact use by our focal species [22]. When possible, we moved plots ≥ 100 m from roads, gas wells, fences, and power lines [25]. Plots that contained ≥ 50% shrub cover were moved to a nearby area with <50% shrub cover when possible [19,26]. Plots were removed from the study when there was no suitable location within 300 m of the randomly selected site. We identified 100 suitable study plots using these methods. We randomly selected 50 of these to survey during the 2017 season and 50 to survey during 2018. In 2018, we repeated field inspections of all plots to ensure plot suitability had not changed during the year.

### Nest surveys

We conducted rope dragging surveys during May and June of 2017 and 2018 to locate longspur nests within plots [27]. Rope dragging was conducted with a weighted 20-m rope and occurred during morning (06:00–10:00 MDT) and evening (17:00–20:00 MDT) hours when adults were more likely to be attending nests [28]. Typically two observers were present for rope drag surveys but occasionally (11/274) three observers were present. To estimate the probability of detecting nests, we surveyed each plot multiple times: 71 plots were surveyed 3 times and the remaining 29 were surveyed twice. To control for possible differences in nest density as the breeding season progressed, we surveyed plots in a random order. However, all replicated surveys were conducted within a 30-day period. To control for the possible diurnal effects in nest detection, we alternated between morning and evening surveys.

For each nest, we recorded the geographic coordinates and marked the site with inconspicuous 25-cm bamboo stakes placed approximately 2 m north and east of the nest to aid in relocating the nest during nest monitoring. Nests discovered incidentally within our study plots were also marked and monitored. For each survey, we recorded the temperature, wind speed, cloud cover, date and time.

We recorded the number of eggs or nestlings in nests at discovery. We conducted subsequent nest visits approximately every 3 days until the nest failed or fledged young. Nests were considered successful if they fledged ≥ 1 young bird, as evidenced from observations of fledglings, parental feeding post-fledging, parental persistent alarm calling in the area of an empty nest, or the presence of fledgling feces in or near nest [29,30]. Nests that did not fledge ≥ 1 young were classified as failed. Nests fail because of abandonment, depredation, or parasitism. Abandonment was identified when the total number of days we observed a nest exceeded the maximum incubation period of the species. Depredation was identified if eggs were suddenly absent from the nest with or without eggshells present, if the nestlings were absent from the nest prior to the earliest possible fledge date, or if the nestlings were gone from the nest at an age when they could have fledged but none of the above evidence of fledging was present. Parasitism was identified if brown-headed cowbirds laid an egg in the nest and the cowbird nestling removed all the host eggs or chicks such that no host chicks fledged. The research protocol was reviewed and exempted by Montana State University’s Institutional Animal Care and Use Committee, as the field study did not involve the capture, handling, housing, transportation, or materially alter the behavior of the animal under study (Animal Welfare Act 9CFR 1.1).

Our protocol provides a conservative estimate of nest survival that may underestimate actual nest success [31]. Accurate estimation is complicated by a wide window of potential fledge dates in the case of our focal species [32]. Longspurs can fledge when their primaries are only half unsheathed and they are unable to fly (J. Pulliam, personal observation). This possibility prevented us from assuming successful fledging based solely on the nestlings age at last check.

### Vegetation surveys

We assessed vegetation conditions at both the plot-scale and the nest-scale. In plots, we sampled random locations until adequate variability was captured to provide an overall estimate of average conditions. We began by generating 15 random points within each of the 100 study plots. We conducted vegetation surveys at five of these points, and calculated the mean and standard deviation of grass cover, using the following equation to determine the sample size needed to accurately represent variation in vegetation measurements within each plot:

$$n={\left(Z_{\alpha }\right)}^{2}{\left(s\right)}^{2}÷{\left(B\right)}^{2}$$

where *n* = the uncorrected sample size, *Z_α_* is the standard normal coefficient calculated for a confidence interval of 90% (1.64), *s* is the sample standard deviation and *B* is the sample mean multiplied by the desired precision (0.15). With the mean and standard deviation along with standard values for *Z_α_* and precision, we calculated the uncorrected sample size. This value was then compared to the table given in Elzinga et al. [33] to get the corrected sample size of vegetation survey points needed. If the corrected sample size was greater than the current number surveyed, we continued to add additional random points until the necessary sample size was reached. We elected to only evaluate appropriate sampling intensity for grass cover because it was the most abundant cover type in each plot and has previously been shown to be a primary habitat condition associated with longspur nests [34]. We acknowledge that it would have been appropriate to apply the formula to each of the 13 vegetation covariates separately and then use the highest number of surveys called for across all covariates, but time limitations prevented us from doing so.

In addition to surveying vegetation at the plot scale we also conducted vegetation surveys at nest sites within three days of fledging or expected fledge date for failed nests. These surveys were identical to the plot scale vegetation surveys except that the point was centered on the nest rather than on a randomly generated point. To account for changes in growth of vegetation between the time when a nest finishes and the time when we conducted plot scale vegetation surveys, each nest vegetation survey was paired with two parallel vegetation surveys at two randomly selected locations within the same survey plot, and all three surveys occurred on the same day.

At each plot-scale sampling site and each nest location, we estimated visual obstruction (VOR), canopy cover, exotic grass cover, litter depth, vegetation height, and standing herbaceous biomass. We recorded VOR to the nearest cm from each of the four cardinal directions at a distance and height of 4 m and 1 m, respectively [35]. We quantified overlapping canopy coverage using 5, 20 × 50 cm Daubenmire frames [36], with one frame centered on the point or nest, and the remaining four placed 0.5 m in each cardinal direction [15]. We estimated overlapping coverages of current growing season grass, residual grass, forbs, shrubs, litter, bare ground, and exotic grass to six percentage bins (0–5, 6–25, 26–50, 51–75, 76–95, and 96–100). We made a distinction between current growing season grass and residual grass from the previous year because longspurs use residual grass in their nest construction. Residual grass cover may also be useful to conceal nests from predators early in the breeding season. Residual grass and litter were distinguished by structure: residual grass maintained an upright stature, similar to what it had when alive, whereas litter was no longer attached to the ground or was lying flat against the ground. When evaluating live or residual grass cover both exotic and native grasses were pooled, but when evaluating exotic grass cover only exotic grasses were included. We measured slope in degrees using a clinometer. We recorded the litter depth, average height of grass, forbs, and shrubs within the frame using a meter stick.

We visually estimated standing herbaceous biomass within each 20 × 50 cm Daubenmire frame in grams. Prior to vegetation sampling each day, we calibrated our visual estimates by clipping and taking the mass of 5–10 frames prior to conducting surveys [37]. To establish the relationship between green and dry herbaceous biomass, we collected all the standing herbaceous vegetation within the northern Daubenmire frame at the first three plot-scale vegetation points in each plot after other vegetation surveys had been completed. These samples were placed in paper sacks and stored it in a shed to air dry. We measured the mass of all samples weekly until they completely dried out, at which point we recorded the dry weight. After all surveys were completed, we calculated a mean difference between estimated green weight and measured green weight for each field observer and applied that calibration to all estimated samples for that observer. We then calculated the mean difference between green weight and dry weight for all samples to convert all estimated green weights to calibrated dry weight.

### Analysis

#### Nest density

Because A) detection probability of nests is likely < 1, and B) the number of nests per plot could have changed within the 30-d survey period due to failure and renesting, we evaluated nest density (nest abundance per 9-ha plot) using the open population N-mixture model of Dail and Madsen [38]. The open N-mixture model (hereafter “DM model”) is a generalization of the single season closed N-mixture mixture model [39] that allows for inference about spatial variation in nest abundance when individual nests are imperfectly detected. The DM model relaxes the closure assumption of Royle [39] and includes explicit parameters (γ = “recruitment”, ω = “survival”) that collectively describe changes in a population over time [38]. Open N-mixture models are often used to estimate local abundances and rates of population change of unmarked animals (e.g., [40,41]), but have not been previously used to estimate nest density. We review the assumptions and suitability of open N-mixture models relative to the estimation of nest density in S1 Appendix in the supplemental materials. We applied all open N-mixture models using the pcountOpen function available in the R package ‘unmarked’ [42]. We evaluated whether populations of nests were open during the survey period by estimating average rates of γ and ω; estimated γ significantly greater than 0 or ω < 1 indicates that the population of nests was not closed.

We evaluated potential overdispersion in the count data by comparing support for two highly-parameterized DM models among two different distributions: the Poisson and negative binomial [43]. The negative binomial distribution had more support (AIC_c_ weight = 0.9) and yielded a dispersion estimate significantly greater than 0 (α = 2.16 ± 0.68SE, *P* < 0.01), indicating data were overdispersed and supporting the use of the negative bionomial mixture. However, diagnostic tests on the negative binomial model in which the tuning parameter K was systematically increased yielded concurrent increases in estimates of slope coefficients and predicted nest abundance, indicating that some parameters were not identifiable using a negative binomial distribution. Similar tests of the Poisson distribution resulted in stable estimates. Therefore, we used the Poisson distribution for all subsequent models and adjusted inferences for overdispersion by using quasi-Akaike’s Information Criterion for finite samples (QAIC_c_) to compare candidate models and adjusting the error of all model estimates using the variance inflation factor ($\hat{c}$) estimated from a parametric bootstrap goodness of fit test calculated using the AICmodavg package [44,45]. The goodness of fit test indicated mild overdispersion (χ^2^ = 316, $\hat{c}$ = 1.23, *P* < 0.05).

Prior to fitting models, we first tested for multicollinearity among vegetation covariates at both the plot and nest-site scales to ensure only uncorrelated variables were included in candidate models (see S2 and S3 Figs in supplemental materials). We calculated the Pearson’s correlation coefficient (*r*) for each combination of vegetation covariates. We considered variables to be correlated if *r* ≥ 0.6. We found significant correlations between biomass and several other vegetation covariates including: VOR (*r* = 0.93), heights of live grass (r = 0.87) and residual grass (*r* = 0.80), and the proportional coverage of exotic grasses (*r* = 0.77). VOR is a commonly used index of herbaceous biomass and an important determinant of grassland bird abundance [34,46–48]. Because biomass is a more common condition informing rangeland management, we retained biomass and removed VOR and the heights of live and residual grass from further analyses [49]. Because we were particularly interested in the effects of exotic grasses on nest density and nest survival, we retained its proportional cover as a covariate but never included both biomass and proportion exotic grass in the same candidate model [50]. We evaluated two candidate model sets, one set containing biomass and one set containing exotic cover with all other uncorrelated variables. Shrub cover was correlated with shrub height (*r* = 0.85); we retained shrub cover as a predictor variable and excluded shrub height due to previously observed associations of grassland birds with shrub coverage [26,46–48,51]. Litter cover was inversely correlated with bare ground cover (*r* = -0.71); litter cover and depth are often associated with bird abundance so we removed bare ground cover from analyses [34]. We also included the within-plot standard deviation of biomass as a potential covariate because heterogeneity of vegetation structure has been demonstrated to be related to bird species diversity [52,53]. Covariates for detection of nests included survey date and time; wind speed, temperature, and cloud cover during the survey, and plot averaged shrub cover. All covariate values were standardized to improve model convergence and allow for direct comparison of covariate effects; however slope coefficients are presented on the real scale unless otherwise noted.

To evaluate the relationships between nest density and plot-scale vegetation variables, we built and evaluated a candidate set of DM models using QAIC_c_. Supported models with large model weights (*w*_*i*_) and QAIC_c_ values ≤ 2 from the best-fit model were considered parsimonious [54]. Measured conditions were previously shown to influence grassland bird detection probability or local abundance [34,55], and we used backward stepwise model selection to identify parsimonious models beginning with submodels of detection probability. Uncorrelated vegetation variables considered in model sets included biomass, proportion forb cover, proportion shrub cover, proportion litter cover, proportion residual grass cover, and slope.

#### Nest survival

We used the nest survival model in program MARK [56] to model the daily nest survival rate (DSR) as a function of vegetation covariates collected at two spatial scales: the nest site and the plot. Program MARK allowed us to use an information theoretic framework to evaluate competing models based on a priori hypotheses about factors influencing nest survival [57]. As we were interested in the effects of vegetation conditions at multiple spatial scales on nest survival, we used a tiered approach for model evaluation. We built and evaluated candidate sets using vegetation data collected at nest sites, a second candidate set using vegetation data collected at the plot-scale, and a final multi-scale analyses including uncorrelated supported vegetation covariates from both spatial extents. Possible factors affecting nest survival at the nest and plot scales included all non-collinear vegetation characteristics. We included a term for survey year in models to account for possible differences in nest survival between years. At the plot-scale we also included the standard deviation (SD) of each vegetation covariate across each plot to evaluate the effects of vegetation variation on nest survival.

Similar to the nest density analysis, we first evaluated multi-collinearity of predictor variables within each spatial scale and only included uncorrelated variables in our candidate models of nest survival (supplementary materials). Because collinearity between biomass and exotic grass cover (*r* = 0.77) prohibited including both terms in the same model, we built and evaluated two sets of candidate models for each species in which one set contained biomass and one set exotic cover. Exotic cover was not correlated with residual grass (*r* = 0.20), forb (*r* = 0.16), shrub (*r* = 0.16), and litter covers (*r* = 0.28; S2 Fig in supplementary materials). For bird species where an effect of exotic grass cover was supported, we used AIC_c_ to compare the top model from the biomass model set and the top model from the exotic cover model set to evaluate which effect had more relative support from the data. To estimate the probability of a nest surviving the entire exposure period, we raised estimated daily survival rate to a power equal to the maximum number of days for a nest to successfully fledge young. The standard error of this estimate was derived using the delta method [58].

#### Nest site conditions vs. plot-scale conditions

We used a correlation analysis to evaluate cross-scale relationships between vegetation conditions measured at local nest sites and average conditions at 9-ha study plots. For study plots that contained at least one longspur nest (71/100), we calculated the average values of each vegetation metric previously identified as having potential influence on longspur nest density or survival. We log- or logit-transformed vegetation measurements if necessary and calculated Pearson’s correlation coefficient (*r*) to assess the relationship between nest and plot-level measures. We deemed an association meaningful if [*r*] > 0.6 and P < 0.05.

#### Plot-scale productivity of longspurs

We conducted an ad hoc analysis to evaluate the relationships between 1) estimated nest survival and nest density and 2) relationship between plot-level longspur productivity and average plot-level herbaceous biomass, deemed an important habitat covariate (see Results and Pulliam et al. 2020). We applied the ranef() function in R package umarked [42] to the most parsimonious model for nest density to estimate the true number of nests, adjusted for detection probability, within each plot. Due to model uncertainty, we used model averaged estimates from our nest survival analysis to predict the average daily survival rate (DSR) of nests within each plot based on the same set of vegetation conditions and calculated the average probability of nest survival within each plot as DSR^30 days. Average plot-scale productivity of longspurs, defined as the expected number of successful nests produced per 9-ha plot, was calculated by multiplying the estimated nest density and average nest survival for each plot. After considering regression diagnostics (Supplemental materials), we evaluated the relationship between estimated productivity and plot-scale biomass using linear regression. The effect of biomass on productivity was considered predictive if the 95% confidence intervals of the effect did not overlap 0 and adjusted *r*^2^ > 0.6.

## Results

### Nest density

We discovered a total of 237 longspur nests during 272 rope drag surveys conducted in 2017 and 2018. We also found an additional 28 nests opportunistically while conducting other activities. The average ± SD number of nests found per plot was 2.65 ± 2.72 and ranged from 0–6 nests. We conducted a total of 776 vegetation surveys at random points within the 100 study plots in addition to the 265 vegetation surveys at each nest site. We found evidence that study plots were not closed to changes in the number of nests during the survey period; although the addition of new nests at plots was negligible (γ = 0.00002 ± 0.002SE), estimated survival was significantly lower than 1.0 (ω = 0.87 ± 0.07SE), collectively indicating a 13% decrease in the number of nests between subsequent surveys at a plot on average.

**Fig 1:**
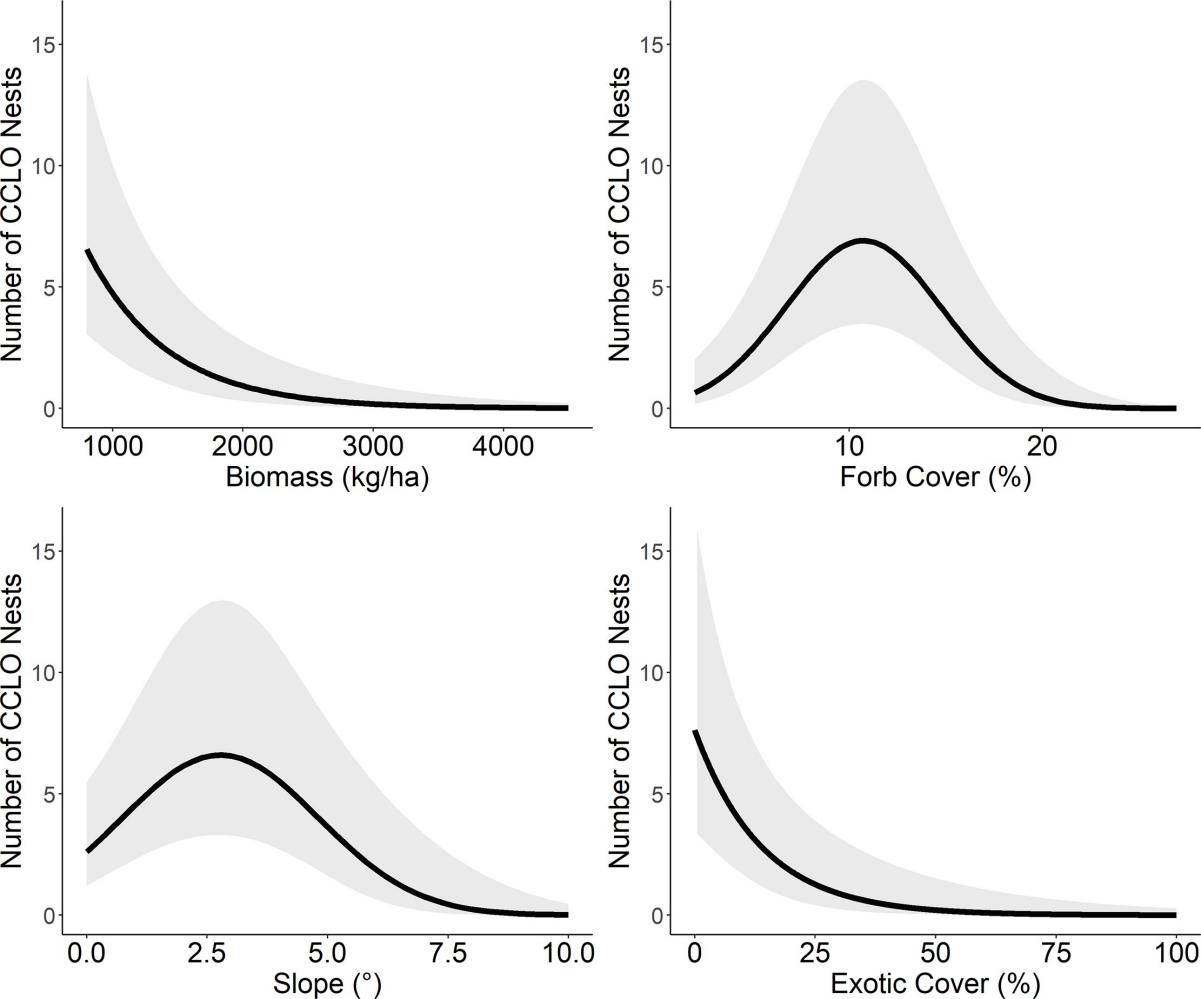
Predicted effect of relevant habitat features on chestnut-collared longspur nest density. Predicted effects of plot-scale herbaceous biomass, forb cover, slope, and exotic grass cover on the number of chestnut-collared longspur nests in Phillips County, Montana during May–June 2017–2018. Shaded areas represent 95% confidence intervals adjusted for overdispersion.

The average probability of detecting a longspur nest was 0.16 ± 0.07SE. Two submodels for detection received approximately equal support (Table 1); both included effects of shrub cover and one also included temperature. However, the effect of temperature overlapped 0 (95%CI = -0.084–0.021) and was considered non-informative. Detection probability declined 16% for every 1% increase in shrub ground cover (β_shrub_ = -0.20 ± 0.06) from 0.38 ± 0.13SE to 0.04 ± 0.02 when shrub cover increased from 0% to 10%. After accounting for spatially-variable detection probability, we found substantial support (QAIC_c_*w* = 0.87) for a single model of longspur nest density that included the effects of plot-scale biomass and quadratic effects of forb cover and slope (Table 1). Longspur nest density was negatively associated with herbaceous biomass within the plot (β_scaled(biomass)_ = -1.65 ± 0.59), where nest density declined 12% for every 100 kg increase in biomass (Fig 1). Nest density exhibited a quadratic relationship with forb cover (β_forb cover_ + β_forb cover_^2^ = 0.49 (0.12SE) - 0.021 (0.006)) and was maximized when forb cover was approximately 11% (Fig 1). Nest density was maximized when slope = ~3° and decreased sharply when slope increased past 3° (β_slope_ + β_slope_^2^ = 0.34 (0.17SE) - 0.07 (0.03); Fig 1).

**Table 1:** Model selection table for nest detection probability and nest density of chestnut-collared longspurs from 272 rope drag surveys of 100 9-ha plots in Phillips County, MT during May–June of 2017 and 2018.

	K	QAICc	ΔQAIC_c_	QAIC_c_ wt
*Detection*				
Shrub Cover	6	581.05	0	0.57
Shrub Cover + Temp	7	581.80	0.75	0.39
Null	5	591.53	10.49	0
*Density with Biomass*				
Biomass + Forb^2^ + Slope^2^	11	533.83	0	0.87
Null	6	691.17	44.21	0
*Density with Exotic Cover*				
Exotic Cover +Forb^2^ + Slope^2^	11	535.47	0	0.83
Null	6	567.25	31.78	0
*Biomass v*. *Exotic Cover*				
Biomass + Forb^2^ + Slope^2^	11	533.83	0	0.99
Exotic Cover +Forb^2^ + Slope^2^	11	543.53	9.71	0.01

The number of parameters (K),QAIC_c_ values, ΔQAIC_c_ values, and model weights (QAIC_c_ wt) are reported. Only models with Akaike weights (ΔAICc) ≤ 2.0 are presented except for the null model and the comparison of exotic cover and biomass models.

A model set evaluating the effects of exotic cover instead of biomass supported effects of exotic cover, forb cover^2^, and slope^2^ on nest density (Table 1). Longspur nest density declined sharply with exotic cover (β = -0.97 ± 0.33 SE; Fig 1). Similar to models that included biomass, nest density exhibited a quadratic relationship with forb cover (β_forb cover_ + β_forb cover_^2^ = 0.20 (0.13SE) - 0.35 (0.11)) and slope (β_slope_ + β_slope_^2^ = 0.06 (0.15SE) - 0.32 (0.13)). The effect of biomass on nest density was more informative than exotic cover; the top model for the set including biomass had virtually all the relative support (Table 1).

**Fig 2:**
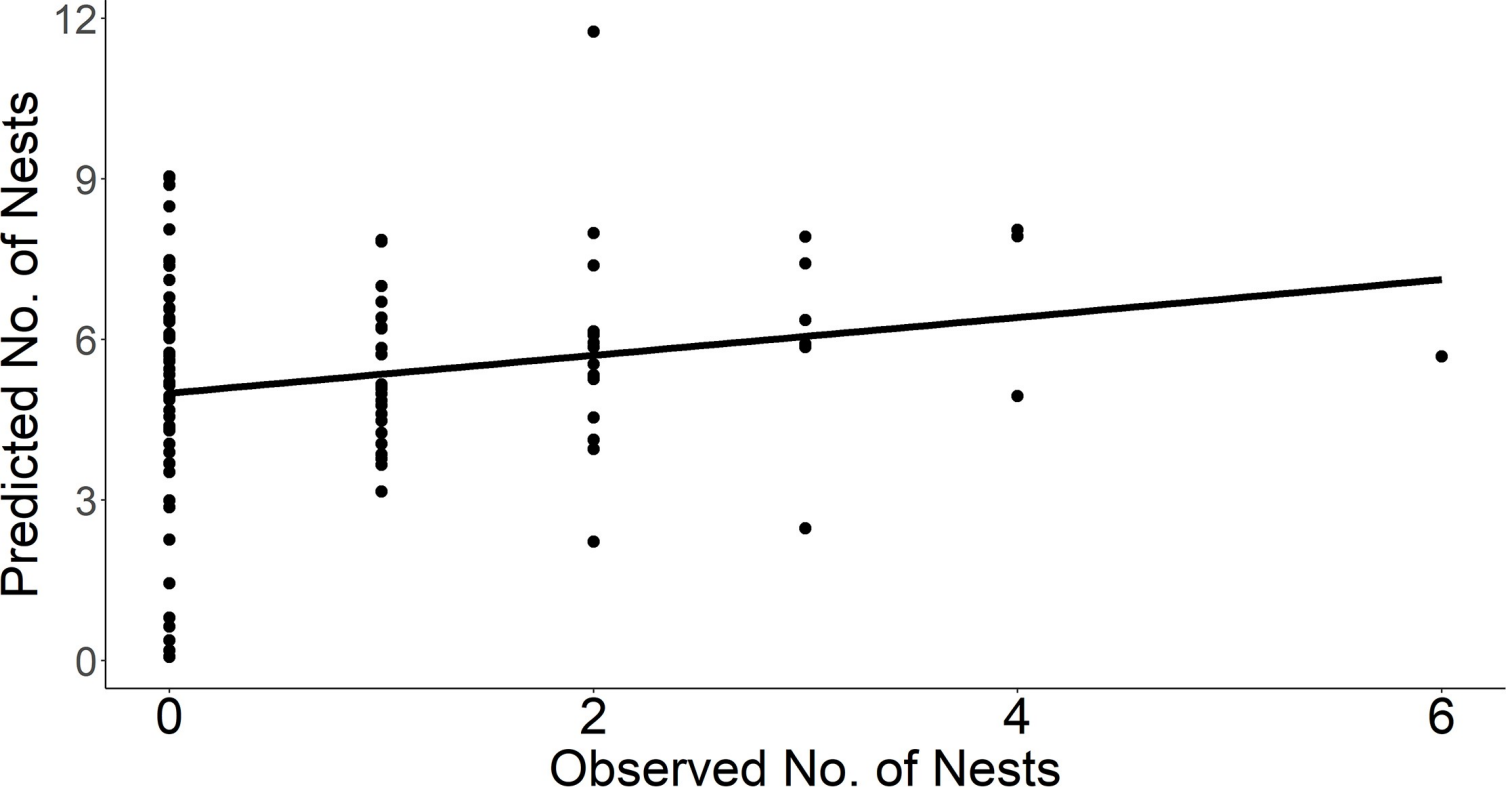
Estimated and observed chestnut-collared longspur nests per study plot. Estimated and observed (raw counts) number of chestnut-collared longspur nests at100, 9-ha plots in Phillips County, MT during May–July of 2017 and 2018. The Pearson’s correlation coefficient indicated a weak positive correlation (*r* = 0.2, *P* = 0.044).

Estimated nest density was consistently greater than the observed density; a single search yielded between 0–90% of the predicted number of nests after adjusting for imperfect detection. There was a weak correlation between the observed and estimated number of nests per plot (*r* = 0.2, P = 0.044; Fig 2).

### Nest survival

**Fig 3:**
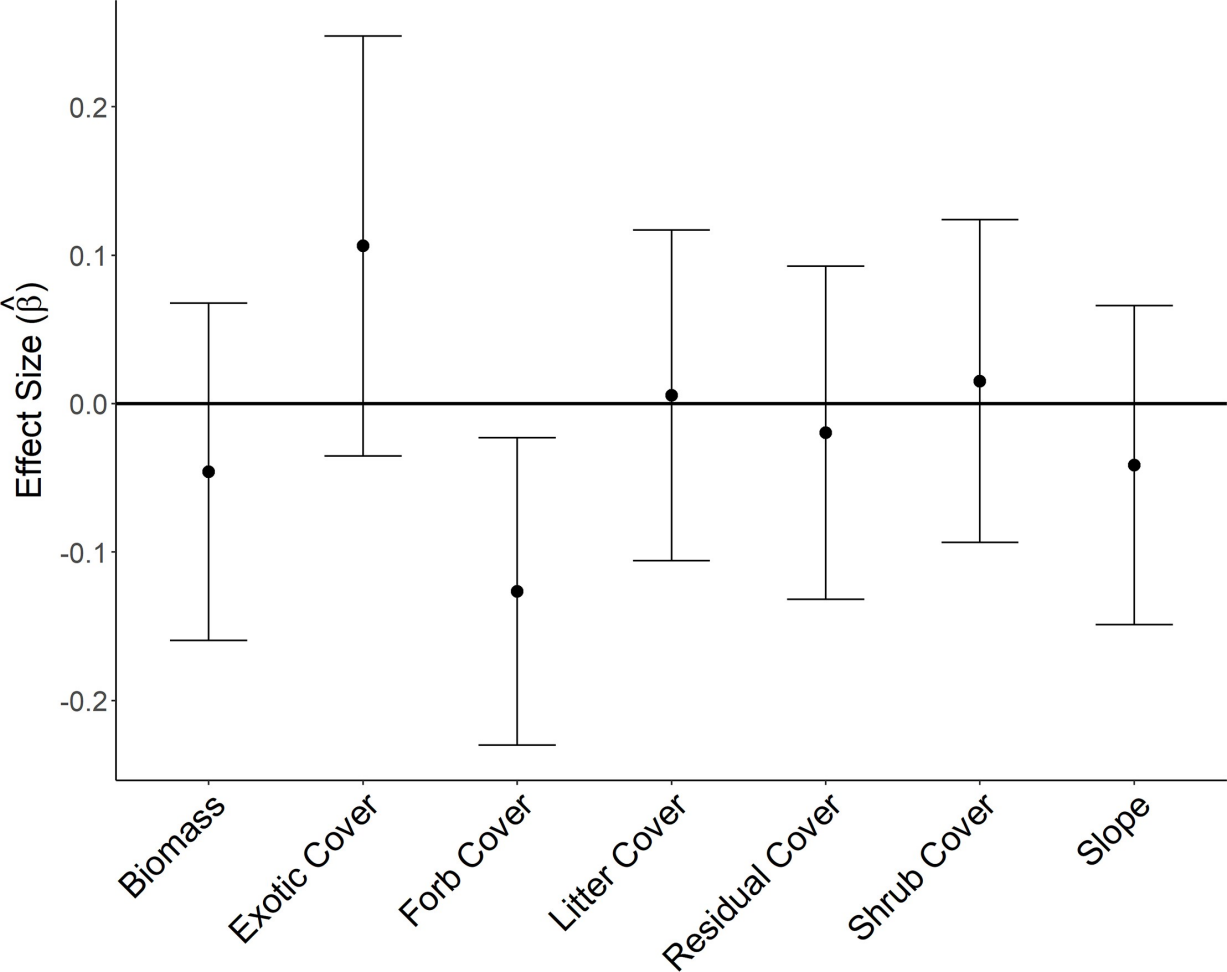
Estimated effect sizes of nest site features on survival of chestnut-collared longspur nests. Estimated effect sizes and 95% confidence intervals from standardized covariates measured at the nest site on daily survival of 263 chestnut-collared longspur nests in 100 9-ha plots in Phillips County, MT, during May–July of 2017 and 2018.

Apparent nest success for longspurs was 29% (76 fledged/263 total nests). Average estimated daily nest survival estimated from the constant (null) model was 0.93 ± 0.01SE, and estimated nest survival for the entire 30-d nest exposure period (DSR^30) was 0.12 ± 0.02.When evaluating vegetation at the nest site, the null model had approximately equal support as the top model (ΔAIC_c_ = 0.91; Table 2), indicating little support for vegetation characteristics at this scale on the daily survival rate of longspur nests. Covariates in equally supported models (ΔAIC_c_ < 2) included survey year, herbaceous biomass, forb cover, litter cover, and exotic cover (Table 2; Fig 3). Notably, a univariate model using forb cover found a significant effect (β = -0.13 ± 0.07 SE). However, this model and all models including forb cover did not have significantly more support than the null model, so we considered the effect non-informative.

**Table 2:** Model selection table for daily survival rate of 263 chestnut-collared longspur nests in 100 9-ha plots in Phillips County, MT, during May–July of 2017 and 2018.

*Nest-site vegetation*	K	AIC_c_	ΔAIC_c_	AIC_c_ Wt
Forb	2	929.16	0	0.13
Year	2	929.29	0.13	0.13
Year + Forb	3	929.35	0.18	0.12
Year + Exotic Cover	3	930.07	0.91	0.09
Null	1	930.08	0.91	0.09
Year + Litter	3	930.43	1.26	0.07
Year + Biomass	3	930.59	1.42	0.07
Exotic Cover	2	930.63	1.47	0.06
*Plot scale vegetation*				
Year	2	929.29	0	0.09
Biomass	2	930.07	0.78	0.06
Null	1	930.08	0.78	0.06
Litter	2	930.27	0.98	0.06
Biomass + Year	3	930.78	1.49	0.04
Biomass + Litter	3	930.97	1.68	0.04
Biomass SD	2	931	1.71	0.04
Exotic + Year	3	931.01	1.71	0.04
Litter SD	2	931.03	1.74	0.04
Exotic SD	2	931.12	1.83	0.04
*Plot scale vegetation* ^b^				
Shrub Cover + Year	3	931.16	1.87	0.04
Residual Grass + Year	3	931.2	1.91	0.03
Litter + Year	3	931.2	1.91	0.03
Litter + Litter SD	3	931.28	1.98	0.03
*Multi-scale Vegetation* ^c^				
Year	2	929.29	0	0.20
Null	1	930.08	0.78	0.13
Nest Forb Cover + Plot Shrub Cover	3	930.79	1.49	0.09
Nest Biomass + Plot Biomass	3	930.85	1.56	0.09
Nest Forb Cover + Plot Residual Grass Cover	3	930.9	1.61	0.09
Nest Exotic Grass Cover + Plot Cover Litter	3	931.02	1.73	0.08

The number of parameters (K), AIC_c_ values, ΔAIC_c_ values, and model weights (AIC_c_Wt) are reported. Vegetation characteristics are evaluated at both the nest-site and plot scale^a^.

^a^ Only models with Akaike weights (ΔAIC_c_ ≥ 2.0 are presented, for full model comparison see Supplementary Materials S2 Table.

^b^ Variables ending in SD indicate the standard deviation of that variable at the plot-scale.

^c^ Variables at the nest-site are denoted with Nest and variables at the plot-scale are denoted with Plot.

**Fig 4:**
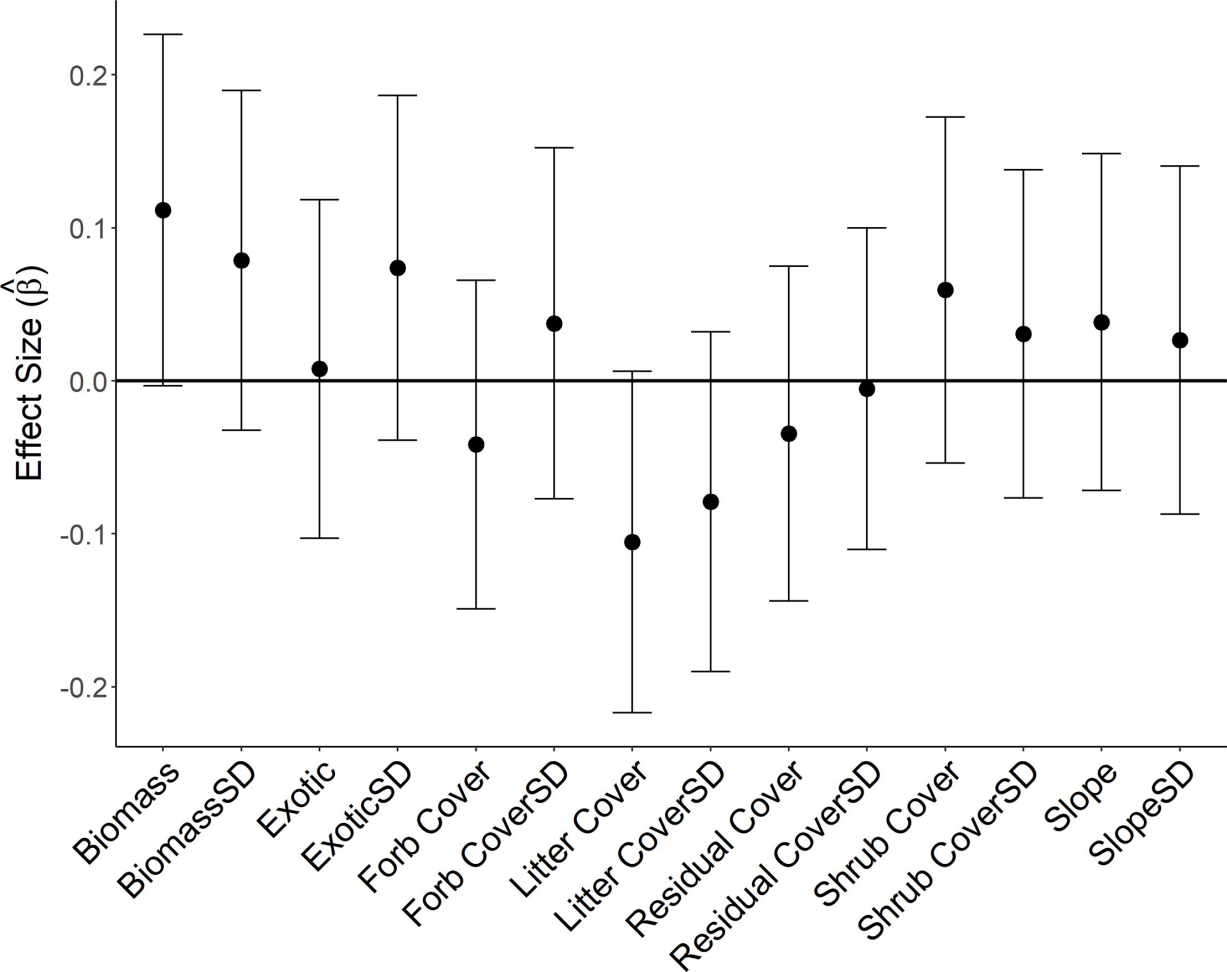
Estimated effect sizes of habitat features on survival of chestnut-collared longspur nests. Estimated effect sizes and 95% confidence intervals from standardized covariates measured at the plot scale on daily survival of 263 chestnut-collared longspur nests in 100 9-ha plots in Phillips County, MT, during May–July of 2017 and 2018. Covariates ending in SD represent the effect of the standard deviation of the covariate at the plot scale.

At the plot-scale, the null model shared similar amount of support as the top model on nest survival (ΔAIC_c_ = 0.78; Table 2). Equally supported models (ΔAIC_c_ < 2) included survey year, herbaceous biomass, litter cover, residual grass cover, shrub cover, and exotic cover, as well as the standard deviations (SD) for biomass, litter cover and exotic cover (Table 2; Fig 4). As we found little evidence of collinearity (*r* < 0.6) among supported covariates across the nest-site and plot-scale, the multi-scale candidate set included all supported terms. In this model set, the null model was also supported (ΔAIC_c_ = 0.78; Table 2) and 95% confidence intervals of covariate effects overlapped 0.

### Nest site vs. plot-scale habitat conditions

We evaluated correlations for 15 vegetation measures collected at both nest sites and randomly across 9-ha study plots. Residual grass height and litter depth were positively correlated across spatial scales (*r* > 0.6, P < 0.05); we did not find significant correlations for other habitat conditions (S1 Table).

### Plot-scale productivity

**Fig 5:**
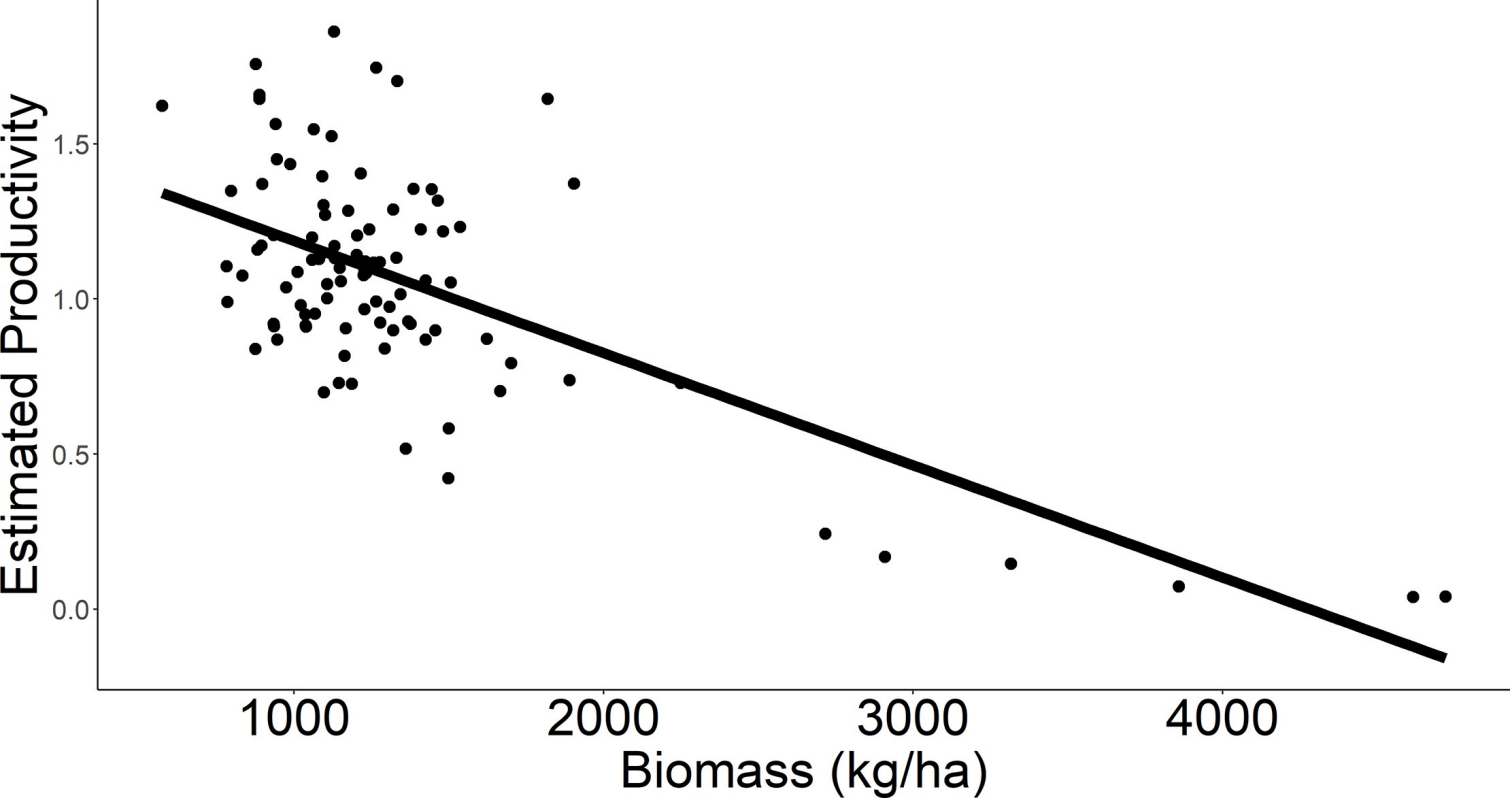
Estimated productivity of chestnut-collared longspurs by plot-scale biomass. Estimated mean productivity (successful nests per 9 ha plot) in relation to average plot-scale herbaceous biomass in Phillips County, MT during May–July of 2017 and 2018.

The support for a null (i.e., constant) model of daily nest survival indicated no relationship between plot-scale nest density and nest survival and suggested that nest density was the most influential component of plot-scale productivity in this system. Longspur productivity declined with herbaceous biomass (β = -0.0004 ± 0.00004; *r*^2^ = 0.53, *P* < 0.001), or 0.04 successful nests per 9-ha plot for every 100 kg/ha increase in biomass (Fig 5). Thus, a management unit (e.g., pasture) 1000 ha in size with an average herbaceous biomass of 1000 kg/ha would be expected to produce approximately 40 more successful nests than a unit of the same size where biomass averages 2000 kg/ha.

## Discussion

### Components of productivity

Effective habitat outcomes must be linked to population productivity to mitigate grassland bird declines. While bird productivity and associated breeding habitat quality are often inferred from nest survival alone, our results suggest that nest density may be a more important driver of population productivity for longspurs, and likely other obligate grassland species. If nest survival represents the population process associated with habitat quality, then habitat predictors of nest density and nest survival should be similar and spatially-explicit estimates of nest density and nest survival should be correlated; however this was not the case. We found that plot-scale habitat conditions influenced nest density, whereas nest survival was unaffected by any measured nest site or plot-scale habitat condition.

Van Horne [59] warned of potential biases that may result by inferring habitat quality from the density of animals (or nests) rather than metrics of population performance, such as nest survival. Disconnects between density and habitat quality may be due to a variety of animal behaviors including site fidelity, maladaptive habitat selection, and social interactions. For example, relatively high densities of animals can occur in sub-optimal habitats when territoriality prevents subdominant individuals from using quality sites or when the historical cues of habitat quality have changed due to human activity [60]. Areas characterized by the former situation are referred to as demographic sinks, whereas those characterized by the latter are termed ecological traps [61,62]. Our results do not support interpretation that areas with high nest density serve as sinks or traps for longspurs because nest survival was unaffected by habitat conditions that varied across the study area. That is, we did not find evidence of maladaptation where breeding densities are higher in areas with depressed reproductive success. Overall, our results are consistent with a growing body of evidence illustrating the importance of factors other than nest survival in determining population productivity (e.g., [63,64]

### Scale mismatch with nest sites

In avian conservation, there are many examples of management recommendations derived from data collected at nest sites as compared to nearby non-used (random) locations. Such recommendations assume that management actions producing outcomes across a larger area (e.g., pasture, patch) will increase the availability of potential nest sites at the fine scale. While there is undoubtedly some relationship in conditions across scales (e.g., a pasture with no shrubs cannot provide shrub-nesting sites), our results show that this relationship is neither straightforward nor linear. Of the 14 vegetation variables we evaluated across plots and at nest sites, only two showed meaningful correlations (r > 0.6) across scales, suggesting that average conditions across a plot did not predict the quality of nest-sites in this system. Further, nest survival did not vary with any measured vegetation attributes, suggesting consistent and effective selection of nest-sites, despite variable plot-scale conditions. Management recommendations based on nest-site and survival data only would have been uninformative in this study.

### Defining habitat outcomes

Our study provides new information on habitat correlates of chestnut-collared longspur productivity that will be useful to outcome-based land management. Specifically, an appropriate amount of standing herbaceous biomass, minimal encroachment by exotic grass species, and adequate availability of forbs were of primary importance for this species. Across the range of variability measured, nest density and resulting population productivity declined sharply with herbaceous biomass. Up to 40 fewer successful nests, or 120–160 fewer fledgling longspurs, would be expected to be produced in a 1000-ha pasture if average standing biomass increased from 1000 to 2000 kg/ha. Our results add to previous work finding lower relative abundance of adults in areas with denser vegetation ([32,49], and suggest that breeding bird densities obtained from point counts occurring after settlement in the spring may adequately index productivity and habitat quality. Interestingly, plot-scale preferences did not translate to improved nest survival. Previous studies similarly found weak or insignificant effects of vegetation structure and composition on nest survival of other grassland birds in the northern Great Plains [16,51]. Future studies aimed at developing habitat targets for management should consider nest density as well as survival.

Exotic grasses have been intentionally introduced throughout the northern Great Plains due to their perceived higher nutritive value to livestock and greater tolerance for drought and cold [65]. Exotic vegetation in the pasture may negatively impact the reproductive output of birds although studies on this effect have produced inconsistent results [13,17]. We found a negative relationship between longspur nest density and cover of exotic grasses, although exotic grass cover was correlated with biomass and effects could not be easily separated. Nevertheless, our results suggest that even low levels (<10% cover) of exotic grass can negatively impact longspur nest density. Avoidance of exotic grasses may occur as a result of effects on other, unstudied, life-history components. For example, a previous study suggested that longspur nestlings in monocultures of exotic crested wheatgrass grew more slowly and fledged at a lower body weight than those in native prairie, likely resulting in lowered recruitment rates [13].

The density of longspur nests was maximized when average forb cover was 10–15%. Forb densities may influence longspur nest site selection via arthropod prey availability. Insects associated with forbs in the orders Coleoptera, Lepidoptera, and especially Orthoptera make up much of the diet of longspurs [32,66]. The selection of nesting areas is likely determined by a tradeoff between security of adults and nests, and the potential to provision young. In addition, optimum values of habitat components are expected to result from niche partitioning among grassland obligates (e.g., Thick-billed longspur, Sprauge’s pipit; [67].

Nest density was highest in areas with modest slopes between 1–3° but most longspurs nested in plots characterized by <5° slope. Breeding habitats have previously been described as ‘flat to rolling’ (e.g., [32,46]), but our results suggest a preference for slightly rolling landscapes. In eastern tallgrass prairies, Frey et al. [68] found a preference among some bird species for certain topographies, but the specific effect that topography has on avian habitat use is not fully understood. Nevertheless, topography can influence plant productivity and diversity which would define available nest sites for grassland birds [69]. Topography may also impact vegetation indirectly by influencing the relative grazing pressure exhibited by livestock in the area [70].

While we found that shrub cover reduced the detection probability of nests, it did not affect nest density. Davis [16] observed that some grassland birds in northern mixed-grass prairies, while avoiding areas with tall dense shrubs, will sometimes place nests close to small shrubs. Despite this observation, he found a positive association between longspur nest placement and distance from the nest to nearest shrub, suggesting these birds avoided shrub cover around their nest. A study of thick-billed longspurs (*Rynchophanes mccownii*) in Colorado, found that half of all nests (14/28) in a moderately grazed pasture were placed beside shrubs and that these nests were 2–3 times more likely to be depredated than those placed distant from shrubs [26]. Only 18% (49/263) of our nests had a shrub within one meter of the nest and our results did not support a negative effect of shrub cover on either nest density or nest survival. One possible reason for this contrast could be the negative effect that shrub cover has on nest detection; it is possible that previous studies observed fewer nests in areas of moderate shrub cover not because there were fewer nests in those areas but because the shrub cover made it more difficult to locate nests. Importantly, shrub cover was low overall across our study area (mean = 3.8% ± 1.6% SD), and may be below thresholds related to nesting impacts on longspurs.

### Estimating nest density

Previously, researchers have addressed biases associated with estimating nest density from an imperfectly-detected sample of nests using modified distance sampling or a time-to-event capture-recapture models [71,72]. Distance sampling has provided imprecise estimates of nest density even for relatively abundant species [73], whereas the model of Péron et al. requires information on the age of nests at time of discovery, which we did not collect due to permit limitations. Additionally, Péron et al. [72] reported numerical instabilities of their model when detection rates were <0.2 and significant bias in estimated nest abundance when nest survival was <0.5; both issues applied to our longspur dataset. Therefore, we evaluated the use of an open N-mixture model [38] applied to replicated independent nest surveys to estimate nest density adjusted for imperfect and spatially-variable detectability of nests. Notably, our results indicated that the probability of detecting a nest was significantly less than 1 and the number of nests within a sampling plot changed even over a relatively short sampling period. Both findings have major implications for research attempting to estimate nest densities for grassland songbirds. The average probability of detecting a longspur nest was 0.16, meaning that naïve estimates of nest density based on raw nest counts were substantially downward biased. While we observed a very weak positive relationship between raw nest counts and estimated nest density, that relationship was significantly less than perfect (0.2:1) and indicated that the bias associated with raw nest counts was spatially variable depending on site-specific habitat conditions (i.e., shrub coverage). Similar to inferring avian abundance from simple counts of birds [49,74], our results indicate that using raw nest counts to compare nest density across sites may result in erroneous inferences about habitat associations with implications for outcome-based management.

The population of longspur nests was not closed during our relatively short sampling period, violating a primary assumption of most nest density analyses. Although recruitment (γ) and survival (ω) parameters of the open N-mixture model may not correspond directly with actual nest initiation rates or nest survival (L. Madsen, Oregon State University, personal communication; Supporting information), they are useful in evaluating whether populations are at equilibrium within a sampling period (i.e., population closure assumption; [38]). The estimated γ within our study plots was near 0 while mean ω = 0.87, suggesting that the number of nests available for detection within a plot declined by approximately 13% ((1 + 0–0.87)*100) between survey events that were separated by 9 days on average. Taken together, analytical methods that assume population closure, such as those based on raw nest counts or closed N-mixture model [39] may be inappropriate for estimating nest abundance and therefore productivity of songbirds. Nevertheless, researchers should ensure assumptions of N-mixture models are adequately addressed through proper study design (see S1 Appendix in supporting information).

## Conclusions

We show that population productivity of longspurs was driven by variability in nest density rather than nest survival. Although nest survival is an important vital rate in avian population dynamics [64] and should not be ignored, our results illustrate the importance of including both density and survival in definition of management outcomes. We caution against use of raw nest counts as a biased index of true nest density, as detectability varies considerably across space and time. Open N-mixture models are a promising method to mitigate detection bias but may have limitations when detection probabilities are low and there is unmodeled heterogeneity in the observation process [75,76].

Additionally, we found that it would be inappropriate in this system to extrapolate vegetation measurements taken at nest-sites to characterize outcomes across broader scales. We encourage researchers to intentionally measure average plot or pasture-scale vegetation attributes with an appropriate sampling method before making management interpretations or designating outcomes at those scales.

We recommend habitat outcomes for breeding longspurs that include maintaining expanses of gently rolling (1–3°) native grassland with 1000–1200 kg/ha of standing herbaceous biomass, minimally invaded by exotic grasses and with high native forb cover of at least 10–15%. In an outcome-based framework, biologists should collaborate closely with rangeland managers skilled in a local context to produce these conditions as possible and appropriate. Although this study did not evaluate specific management actions, grazing is a widespread, compatible land use and is likely to be a primary tool influencing habitat outcomes for longspurs. Grazing management can be designed in the local context to manipulate biomass (e.g. [77,78]), reduce exotic species invasion (e.g. [77]) and increase forb cover (e.g. [79]).

## Supporting information

S1 Fig Study area. Study plots on Bureau of Land Management (BLM), National Wildlife Refuge (NWR), Montana State Trust (ST) land, and private land in Phillips County, Montana. Inserts show a closeup of some plots in the northern and southern parts of the county.
(TIF)

S2 Fig Collinearity of plot-scale vegetation metrics. Results of pair-wise collinearity comparison for all vegetation metrics at the 9-ha plot scale. Metrics include visual obstruction reading (VOR), slope, live grass cover (Grass_Cover), live grass height (Grass_Ht), residual grass cover (Resid_Cover), residual grass height (Resid_Ht), bare ground cover (BG), litter cover (Litter_Cover), forb cover (Forb_Cover), forb height (Forb_Ht), shrub cover (Shrub_Cover), shrub height (Shrub_Ht), exotic grass cover (Exotic_Cover) litter depth (Litter_Depth), herbaceous standing biomass (Biomass), and the standard deviation of herbaceous standing biomass (SD_Bio). Pearson’s correlation coefficient are given above the diagonal.
(TIF)

S3 Fig Collinearity of nest site vegetation metrics. Results of pair-wise collinearity comparison for all vegetation metrics at the nest-site scale. Metrics include visual obstruction reading (VOR), slope, live grass cover (Grass_Cover), live grass height (Grass_Ht), residual grass cover (Resid_Cover), residual grass height (Resid_Ht), bare ground cover (BG), litter cover (Litter_Cover), forb cover (Forb_Cover), forb height (Forb_Ht), shrub cover (Shrub_Cover), shrub height (Shrub_Ht), exotic grass cover (Exotic_Cover) litter depth (Litter_Depth), herbaceous standing biomass (Biomass), and the standard deviation of herbaceous standing biomass (SD_Bio). Pearson’s correlation coefficient are given above the diagonal.
(TIF)

S4 Fig Collinearity of nest site vegetation metrics. Relationship between vegetation conditions measured at the nest sites of 263 chestnut-collared longspur nests and across the study plot (9 ha). Vegetation surveys took place May–July 2017 and 2018 in Phillips County, Montana.
(TIF)

S1 Table Habitat conditions. Average ± standard deviation vegetation conditions across land ownership types Bureau of Land Management (BLM), private land (PL), Montana state-trust land (ST), and National Wildlife Refuge land (NWR), for 100 9-ha plots in Phillips County, MT in May–July 2017 and 2018.
(DOCX)

S2 Table Model selection for nest survival. AIC_c_ model selection table for daily survival rate of 263 Chestnut-collared Longspur nests in 100 9-ha plots in Phillips County, MT, during May–July of 2017 and 2018. Vegetation characteristics are evaluated at both the nest-site and plot scale^a^.
(DOCX)

S3 Table Correlation of habitat conditions. Pearson’s correlation coefficients of vegetation conditions at 71, 9-ha study plots and at the nest sites of 263 Chestnut-collared Longspur nests within those plots in Phillips county Montana during May—July 2017 and 2018, * indicates *P* < 0.05.
(DOCX)

S1 Appendix Assumptions of the open N-mixture model as applied to the estimation of avian nest density. (DOCX)
