# Supplementary figures and images for: Nest density drives productivity in chestnut-collared longspurs: Implications for grassland bird conservation
Source: PLoS One. 2021 Aug 24;16(8):e0256346. doi: 10.1371/journal.pone.0256346 (PMC8384174; doi:10.1371/journal.pone.0256346)

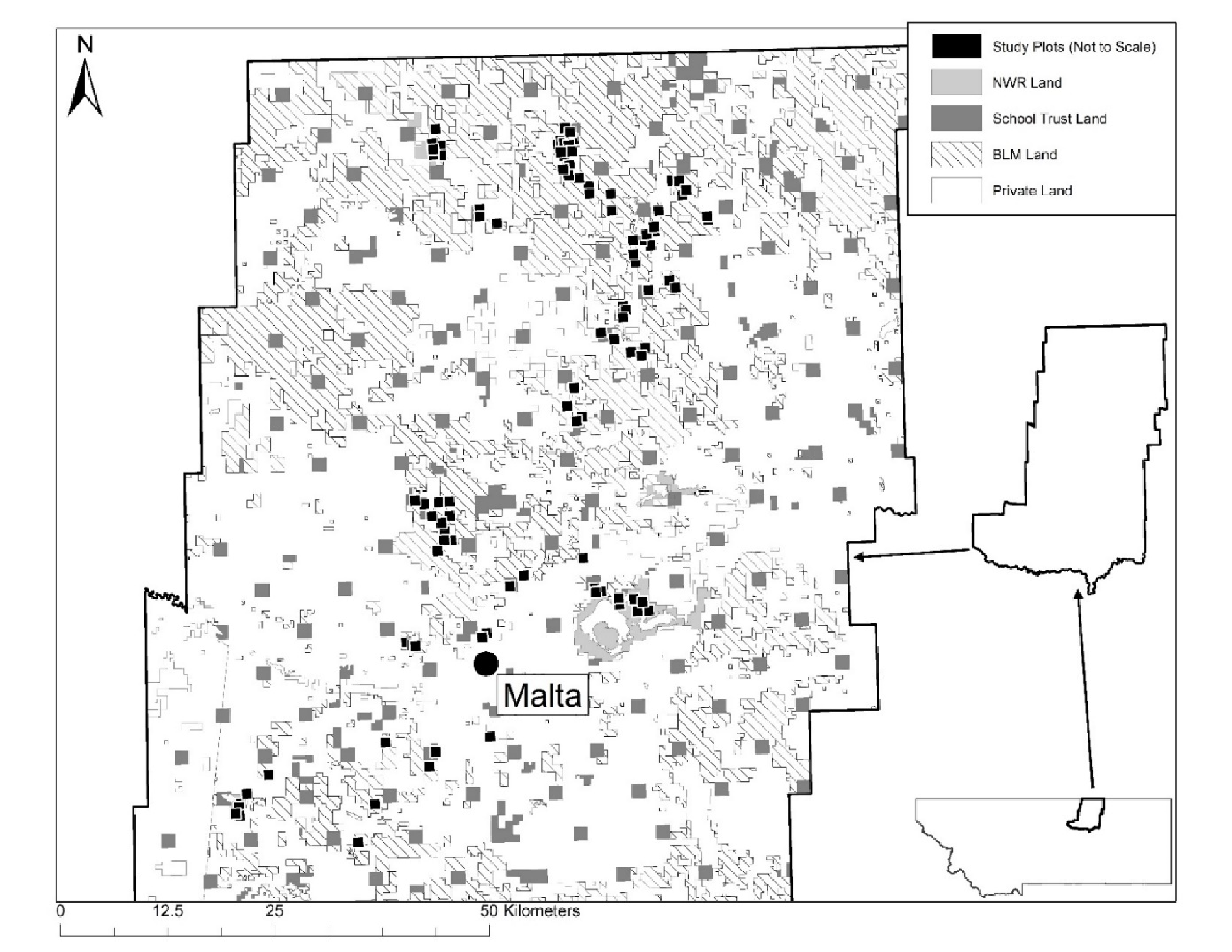

Supplement: S1 Fig — Study plots on Bureau of Land Management (BLM), National Wildlife Refuge (NWR), Montana State Trust (ST) land, and private land in Phillips County, Montana. Inserts show a closeup of some plots in the northern and southern parts of the county. (TIF) [file pone.0256346.s001.tif]

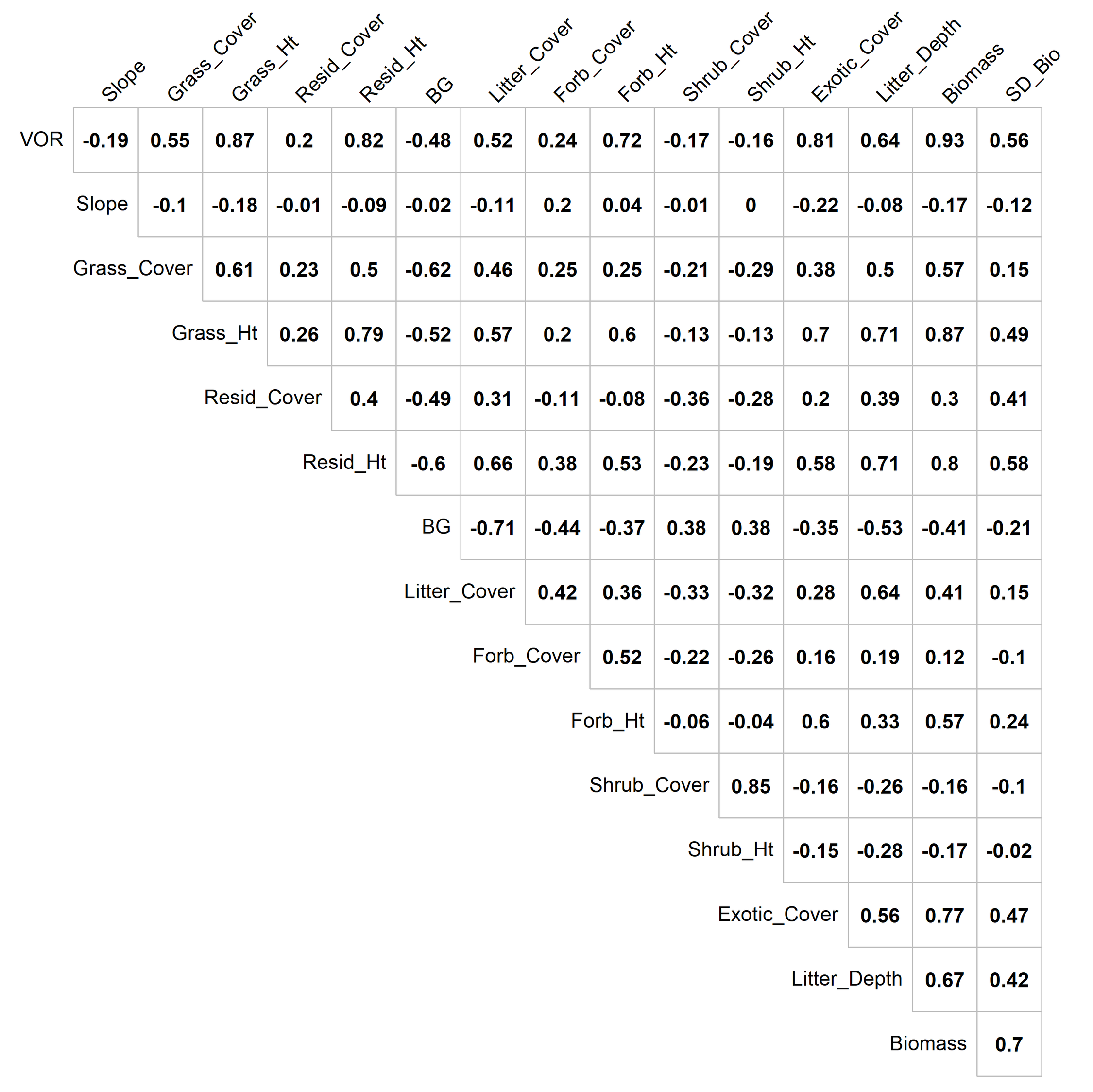

Supplement: S2 Fig — Results of pair-wise collinearity comparison for all vegetation metrics at the 9-ha plot scale. Metrics include visual obstruction reading (VOR), slope, live grass cover (Grass_Cover), live grass height (Grass_Ht), residual grass cover (Resid_Cover), residual grass height (Resid_Ht), bare ground cover (BG), litter cover (Litter_Cover), forb cover (Forb_Cover), forb height (Forb_Ht), shrub cover (Shrub_Cover), shrub height (Shrub_Ht), exotic grass cover (Exotic_Cover) litter depth (Litter_Depth), herbaceous standing biomass (Biomass), and the standard deviation of herbaceous standing biomass (SD_Bio). Pearson’s correlation coefficient are given above the diagonal. (TIF) [file pone.0256346.s002.tif]

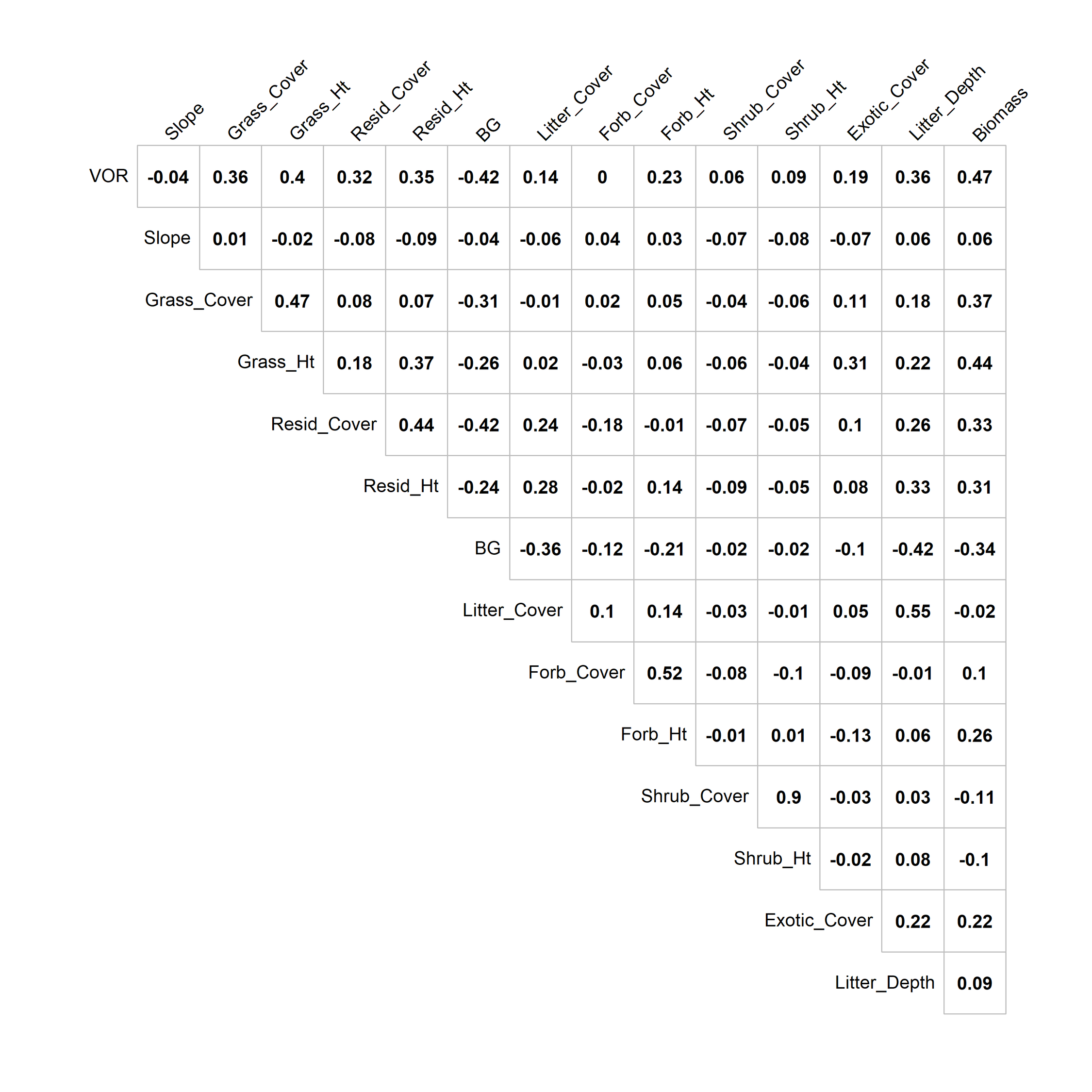

Supplement: S3 Fig — Results of pair-wise collinearity comparison for all vegetation metrics at the nest-site scale. Metrics include visual obstruction reading (VOR), slope, live grass cover (Grass_Cover), live grass height (Grass_Ht), residual grass cover (Resid_Cover), residual grass height (Resid_Ht), bare ground cover (BG), litter cover (Litter_Cover), forb cover (Forb_Cover), forb height (Forb_Ht), shrub cover (Shrub_Cover), shrub height (Shrub_Ht), exotic grass cover (Exotic_Cover) litter depth (Litter_Depth), herbaceous standing biomass (Biomass), and the standard deviation of herbaceous standing biomass (SD_Bio). Pearson’s correlation coefficient are given above the diagonal. (TIF) [file pone.0256346.s003.tif]

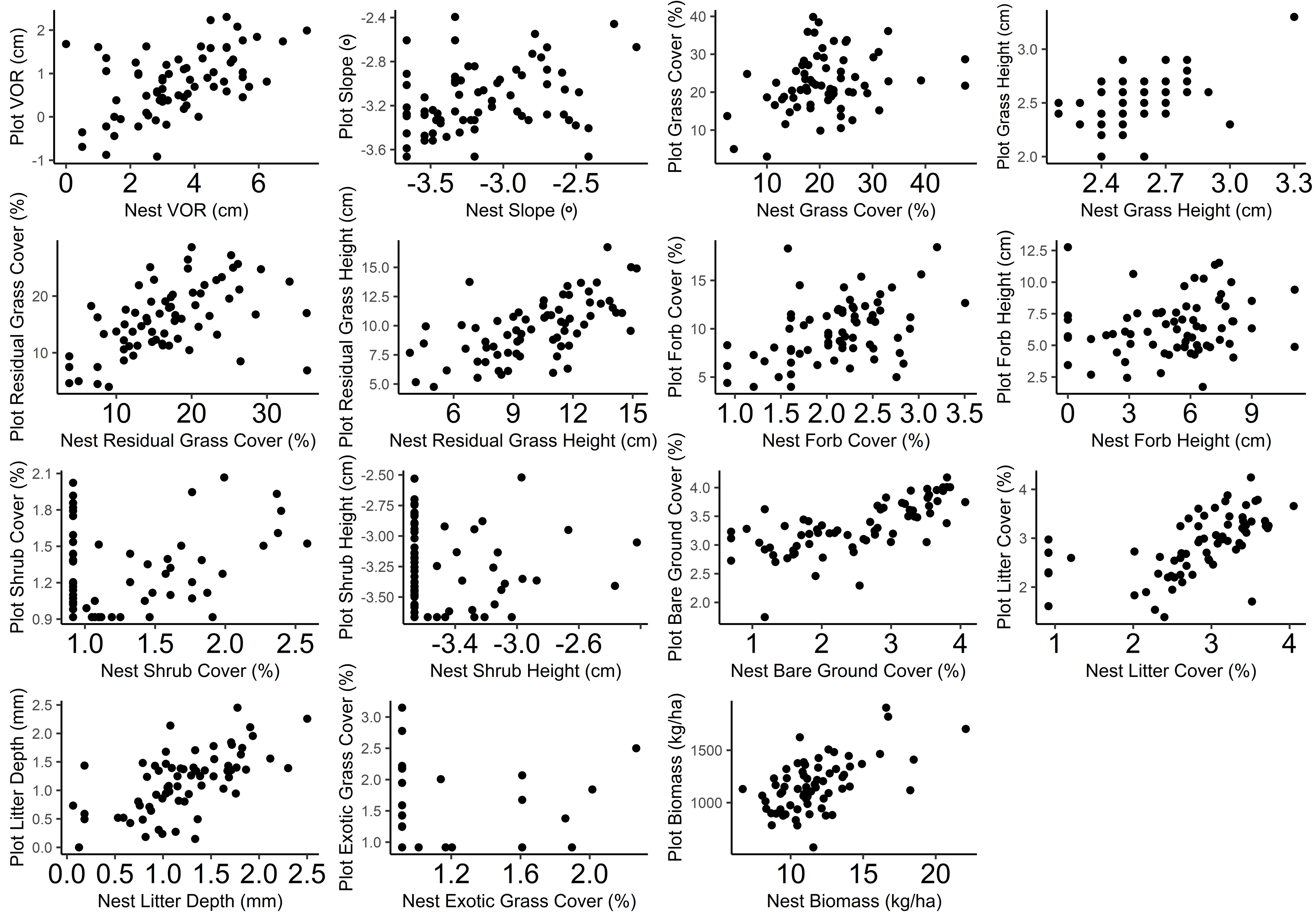

Supplement: S4 Fig — Relationship between vegetation conditions measured at the nest sites of 263 chestnut-collared longspur nests and across the study plot (9 ha). Vegetation surveys took place May–July 2017 and 2018 in Phillips County, Montana. (TIF) [file pone.0256346.s004.tif]
