# Supplementary material for: Nest density drives productivity in chestnut-collared longspurs: Implications for grassland bird conservation
Source: PLoS One. 2021 Aug 24;16(8):e0256346. doi: 10.1371/journal.pone.0256346 (PMC8384174; doi:10.1371/journal.pone.0256346)
Supplement: S1 Table — Average ± standard deviation vegetation conditions across land ownership types Bureau of Land Management (BLM), private land (PL), Montana state-trust land (ST), and National Wildlife Refuge land (NWR), for 100 9-ha plots in Phillips County, MT in May–July 2017 and 2018. (DOCX) [file pone.0256346.s005.docx]

| **S1 TABLE.** **Habitat conditions.** Average ± standard deviation vegetation conditions across land ownership types Bureau of Land Management (BLM), private land (PL), Montana state-trust land (ST), and National Wildlife Refuge land (NWR), for 100 9-ha plots in Phillips County, MT in May – July 2017 and 2018. | | | | |
| --- | --- | --- | --- | --- |
|  | BLM | PL | ST | NWR |
| VOR | 2.68 ± 1.72 | 2.14 ± 1.29 | 5.12 ± 3.28 | 18.56 ± 13.19 |
| Slope (°) | 2.3 ± 2.11 | 2.53 ± 2.49 | 2.03 ± 1.84 | 0.91 ± 1.26 |
| Grass Cover (%) | 20.99 ± 7.73 | 26 ± 5.24 | 25.69 ± 9.56 | 32.81 ± 18.44 |
| Grass Height (cm) | 12.98 ± 3.28 | 13.89 ± 1.23 | 15.39 ± 4.43 | 28.15 ± 10.23 |
| Residual Cover (%) | 15.46 ± 5.68 | 18.43 ± 5.26 | 18.46 ± 8.10 | 21.55 ± 11.95 |
| Residual Ht (cm) | 9.58 ± 2.44 | 12.37 ± 1.72 | 12.55 ± 5.64 | 18.52 ± 8.09 |
| Bare Ground (%) | 32.19 ± 13.50 | 19.46 ± 4.52 | 25.46 ± 13.78 | 12.61 ± 15.2 |
| Litter Cover (%) | 18.03 ± 0.76 | 30.17 ± 9.29 | 27.73 ± 19.35 | 43.07 ± 22.14 |
| Forb Cover (%) | 9.66 ± 3.41 | 12.6 ± 6.14 | 10.11 ± 4.74 | 10.34 ± 6.08 |
| Forb Height (cm) | 6.51 ± 2.22 | 6.59 ± 2.37 | 7.03 ± 2.43 | 13.08 ± 9.48 |
| Shrub Cover (%) | 3.91 ± 1.68 | 3.97 ± 1.90 | 3.98 ± 1.60 | 3.12 ± 1.28 |
| Shrub Height (cm) | 1.69 ± 1.75 | 1.43 ± 1.64 | 1.85 ± 1.62 | 0.79 ± 1.7 |
| Exotic Cover (%) | 4.46 ± 4.71 | 2.6 ± 0.24 | 5.48 ± 5.33 | 32 ± 32.18 |
| Litter Depth (mm) | 3.28 ± 1.83 | 3.36 ± 0.91 | 4.98 ± 3.00 | 13.65 ± 11.75 |
| Biomass (kg/ha) | 1134 ± 229 | 1231 ± 165 | 1397 ± 320 | 2633 ± 1250 |
