# Supplementary material for: Nest density drives productivity in chestnut-collared longspurs: Implications for grassland bird conservation
Source: PLoS One. 2021 Aug 24;16(8):e0256346. doi: 10.1371/journal.pone.0256346 (PMC8384174; doi:10.1371/journal.pone.0256346)
Supplement: S2 Table — AICc model selection table for daily survival rate of 263 Chestnut-collared Longspur nests in 100 9-ha plots in Phillips County, MT, during May–July of 2017 and 2018. Vegetation characteristics are evaluated at both the nest-site and plot scalea. (DOCX) [file pone.0256346.s006.docx]

| **S2 Table.** **Model selection for nest survival.** AIC_c_ model selection table for daily survival rate of 263 Chestnut-collared Longspur nests in 100 9-ha plots in Phillips County, MT, during May – July of 2017 and 2018. Vegetation characteristics are evaluated at both the nest-site and plot scale^a^ | | | | |
| --- | --- | --- | --- | --- |
| *Nest-site vegetation* | *k* | AIC_c_ | ΔAIC_c_ | AIC_c_ Wt |
| Forb | 2 | 929.16 | 0 | 0.13 |
| Year | 2 | 929.29 | 0.13 | 0.13 |
| Year + Forb | 3 | 929.35 | 0.18 | 0.12 |
| Year + Exotic | 3 | 930.07 | 0.91 | 0.09 |
| Null | 1 | 930.08 | 0.91 | 0.09 |
| Year + Litter | 3 | 930.43 | 1.26 | 0.07 |
| Year + Biomass | 3 | 930.59 | 1.42 | 0.07 |
| Exotic | 2 | 930.63 | 1.47 | 0.06 |
| Year + Residual Grass | 3 | 931.28 | 2.11 | 0.05 |
| Year + Shrub | 3 | 931.3 | 2.13 | 0.05 |
| Linear Biomass | 2 | 931.75 | 2.59 | 0.04 |
| Residual Grass | 2 | 932.02 | 2.85 | 0.03 |
| Shrub | 2 | 932.04 | 2.88 | 0.03 |
| Litter | 2 | 932.07 | 2.91 | 0.03 |
| Biomass + Residual Grass | 3 | 933.74 | 4.58 | 0.01 |
| *Plot scale vegetation* |  |  |  |  |
| Year | 2 | 929.29 | 0 | 0.09 |
| Biomass | 2 | 930.07 | 0.78 | 0.06 |
| Null | 1 | 930.08 | 0.78 | 0.06 |
| Litter | 2 | 930.27 | 0.98 | 0.06 |
| Biomass + Year | 3 | 930.78 | 1.49 | 0.04 |
| Biomass + Litter | 3 | 930.97 | 1.68 | 0.04 |
| Biomass SD | 2 | 931 | 1.71 | 0.04 |
| Exotic + Year | 3 | 931.01 | 1.71 | 0.04 |
| Litter SD | 2 | 931.03 | 1.74 | 0.04 |
| Exotic SD | 2 | 931.12 | 1.83 | 0.04 |
| *Plot scale vegetation*^b^ | *k* | AIC_c_ | ΔAIC_c_ | AIC_c_ Wt |
| Shrub Cover + Year | 3 | 931.16 | 1.87 | 0.04 |
| Residual Grass + Year | 3 | 931.2 | 1.91 | 0.03 |
| Litter + Year | 3 | 931.2 | 1.91 | 0.03 |
| Litter + Litter SD | 3 | 931.28 | 1.98 | 0.03 |
| Shrub Cover | 2 | 931.49 | 2.2 | 0.03 |
| Biomass + Residual Grass | 3 | 931.57 | 2.28 | 0.03 |
| Forb Cover | 2 | 931.77 | 2.48 | 0.03 |
| Slope | 2 | 931.83 | 2.53 | 0.03 |
| Forb SD | 2 | 931.86 | 2.56 | 0.03 |
| Residual Grass | 2 | 931.87 | 2.58 | 0.02 |
| Shrub SD | 2 | 931.91 | 2.62 | 0.02 |
| Slope SD | 2 | 931.96 | 2.67 | 0.02 |
| Biomass + Biomass SD | 3 | 932.04 | 2.75 | 0.02 |
| Exotic Grass Cover | 2 | 932.07 | 2.78 | 0.02 |
| Residual Grass SD | 2 | 932.07 | 2.78 | 0.02 |
| Exotic Grass Cover + Litter | 3 | 932.21 | 2.91 | 0.02 |
| Exotic Grass Cover + Exotic Grass SD | 3 | 933.12 | 3.83 | 0.01 |
| Shrub Cover + Shrub Cover SD | 3 | 933.33 | 4.04 | 0.01 |
| Forb Cover + Forb SD | 3 | 933.64 | 4.34 | 0.01 |
| Slope + Slope SD | 3 | 933.64 | 4.34 | 0.01 |
| Residual Grass + Residual Grass SD | 3 | 933.77 | 4.48 | 0.01 |
| Exotic Grass Cover + Residual Grass | 3 | 933.86 | 4.57 | 0.01 |
| *Multi-scale Vegetation*^c^ |  |  |  |  |
| Year | 2 | 929.29 | 0 | 0.20 |
| Null | 1 | 930.08 | 0.78 | 0.13 |
| Nest Forb Cover + Plot Shrub Cover | 3 | 930.79 | 1.49 | 0.09 |
| Nest Biomass + Plot Biomass | 3 | 930.85 | 1.56 | 0.09 |
| *Multi-scale Vegetation*^c^ | *k* | AIC_c_ | ΔAIC_c_ | AIC_c_ Wt |
| Nest Forb Cover + Plot Residual Grass Cover | 3 | 930.9 | 1.61 | 0.09 |
| Nest Exotic Grass Cover + Plot Cover Litter | 3 | 931.02 | 1.73 | 0.08 |
| Nest Exotic Grass Cover + Plot Exotic Grass SD | 3 | 931.54 | 2.25 | 0.06 |
| Nest Litter Cover + Plot Litter Cover | 3 | 931.8 | 2.51 | 0.06 |
| Nest Biomass + Plot Biomass SD | 3 | 932.47 | 3.18 | 0.04 |
| Nest Biomass + Plot Biomass + Plot Shrub Cover | 4 | 932.58 | 3.29 | 0.04 |
| Nest Exotic Grass Cover + Plot Exotic Grass Cover | 3 | 932.61 | 3.31 | 0.04 |
| Nest Exotic Grass Cover + Plot Litter Cover + Plot Exotic Grass Cover | 4 | 932.75 | 3.46 | 0.03 |
| Nest Litter Cover + Plot Litter SD | 3 | 933.03 | 3.74 | 0.03 |
| Nest Exotic + Plot Residual + Plot Exotic Grass | 4 | 934.51 | 5.21 | 0.01 |
| ^a^ Only models with Akaike weights (AIC_c_ Wt) ≥ 0.01 are presented  ^b^ Variables ending in SD indicate the standard deviation of that variable at the plot-scale  ^c^ Variables at the nest-site are denoted with Nest and variables at the plot-scale are denoted with Plot |  |  |  |  |
|  |  |  |  |  |
|  |  |  |  |  |
