# Supplementary material for: Nest density drives productivity in chestnut-collared longspurs: Implications for grassland bird conservation
Source: PLoS One. 2021 Aug 24;16(8):e0256346. doi: 10.1371/journal.pone.0256346 (PMC8384174; doi:10.1371/journal.pone.0256346)
Supplement: S3 Table — Pearson’s correlation coefficients of vegetation conditions at 71, 9-ha study plots and at the nest sites of 263 Chestnut-collared Longspur nests within those plots in Phillips county Montana during May—July 2017 and 2018, * indicates P < 0.05. (DOCX) [file pone.0256346.s007.docx]

| **S3 TABLE.**  **Correlation of habitat conditions.** Pearson’s correlation coefficients of vegetation conditions at 71, 9-ha study plots and at the nest sites of 263 Chestnut-collared Longspur nests within those plots in Phillips county Montana during May - July 2017 and 2018, * indicates *P* < 0.05. | |
| --- | --- |
| Vegetation Metric | *r* |
| VOR | 0.49* |
| Slope | 0.31* |
| Grass Cover | 0.29* |
| Grass Height | 0.54* |
| Residual Grass Cover | 0.5* |
| Residual Grass Height | 0.62* |
| Bare Ground Cover | 0.69* |
| Litter Cover | 0.55* |
| Forb Cover | 0.44* |
| Forb Height | 0.17 |
| Shrub Cover | 0.32* |
| Shrub Height | -0.01 |
| Exotic Cover | 0.31* |
| Litter Depth | 0.62* |
| Biomass | 0.56* |
