# Supplementary material for: Nest density drives productivity in chestnut-collared longspurs: Implications for grassland bird conservation
Source: PLoS One. 2021 Aug 24;16(8):e0256346. doi: 10.1371/journal.pone.0256346 (PMC8384174; doi:10.1371/journal.pone.0256346)
Supplement: S1 Appendix — (DOCX) [file pone.0256346.s008.docx]

**S1 Appendix. Assumptions of the open N-mixture model as applied to the estimation of avian nest density**

The open N-mixture model a generalization of the single season closed N-mixture mixture model (Royle 2004) that allows for inference about spatial variation in nest abundance when individual nests are imperfectly detected. The model relaxes the closure assumption of Royle (2004) and includes explicit parameters (γ = “recruitment”, ω = “survival”) that collectively describe changes in a population over time (Dail and Madsen 2011). Open N-mixture models were developed and have been previously used to estimate local abundances and rates of population change of unmarked animals (e.g., Hostetler and Chandler 2015, Kidwai et al., 2019), but have not been used to estimate nest density prior to this study. Researchers recently developed a time-to-event capture-recapture model to simultaneously estimates detection-corrected nest density and nest survival (Péron et al. 2014); the model however requires large sample sizes of nests, relatively high nest survival rates, and that the age of nests at time of discovery is known. The open N-mixture model may provide an alternative to estimating nest density when these conditions are not met.

Before applying the open N-mixture model to the estimation of nests, an evaluation of its assumptions is necessary (Knape and Korner-Nievergelt 2015). Assumptions of the closed N-mixture model (Royle 2004) also relevant to the generalized open N-mixture model of Dail and Madsen (2011) are:

1. **Sites are independent,**
2. **There are no false positives and that a single nest is never counted as two unique nests,**
3. **Distributional assumptions (Poisson for the initial abundance component, and Binomial for the observation process),**
4. **No unmodeled heterogeneity in detection probability.**
   - - 1. **Sites are independent** – The first assumption can be met by proper design to ensure that study plots are independent, that is nests are not counted at multiple plots and, more generally, the abundance of nests at one plot does not influence the abundance of nests at another plot. Ideally, study plots would be separated by a distance that ensures a lack of spatial correlation in nest density. In our study, the average nearest distance between survey plots was 855 m (range = 25–6231 m). Realistically, nesting habitats are almost always clustered in space meaning that study plots in proximity are more likely to share habitat features that those farther apart. Thus, adequate sample sizes of plots and nests needed for reasonable precision will likely result in some spatial correlation in nest density for grassland birds if covariates responsible for the spatial correlation are not explicitly incorporated into the model. We believe the assumption of site independence is met by our dataset because 1) nests are immobile (i.e., they cannot be counted at two study plots), and 2) we evaluated and incorporated habitat covariates into our models that we hypothesized explain any spatial correlation.
       2. **No false positives** – This assumption implies that all nests included in the sample were those of chestnut-collared longspurs (CCLO) and that individual nests are not counted as two unique nests. Identification of nests to species was conducted by experienced field ornithologists using field guides and confirmed via identification of an attending adult. A single nest could not be counted as two unique nests since nests do not move and were uniquely marked at time of discovery.
       3. **Distributional assumptions** – The DM model incorporates a demographic model for ‘recruitment’ and ‘survival’ applied to a vector of initial local abundances estimated using a Poisson distribution (or negative binomial and zero-inflated Poisson for special cases of overdispersion) and a binomial detection model. The Poisson is an intuitive distribution describing the spatial variation of local nest abundance at a collection of sites where the number of nests are positive integers (0, 1, 2,…; McCullagh and Nelder 1989, Kéry and Schaub 2012). A potential limitation is that the variance of a Poisson random variable is identical to the mean (i.e., the expected count under the model). The mean = variance assumption is often violated in nature, especially for rare organisms, when the variance of counts is larger than the mean – a situation known as overdispersion. Overdispersion can arise when important covariates are not incorporated or when observation units are not independent. Although overdispersion will not affect the estimated means, it can lead to downward-biased estimates of precision and liberal inferences regarding effects of covariates (Kéry and Schaub 2012). The DM model addresses this issue by allowing the specification of two other distributions, the negative binomial and zero-inflated Poisson, that incorporate additional parameters for variance (Royle 2004, Joseph et al. 2009). Formal evaluations for overdispersion are available and advised (Fiske and Chandler 2011). A binomial distribution assumption for the probability of detection is natural as well, as it formalizes a 0/1 (e.g., coin flip) detection processes. The binomial assumption underlies most capture-recapture models.
       4. **No unmodeled heterogeneity in detection probability** – Unmodeled heterogeneity in detection probability across survey periods and sites can result in biased estimates of nest density, and emphasizing the need for proper evaluation of site and survey covariates on detection probability that describe spatial and temporal causes of detection probability (Kéry and Schaub 2012) – indeed identification of site and survey conditions that determine spatiotemporal variation in detection and abundance was a primary objective or our study. In addition, the probability of detecting a nest is assumed to be constant for individual nests within a plot at survey time *j*. That is, the model does not accommodate individual heterogeneity in detection probability among nests that is not determined by habitat or survey conditions. This assumption has implications for the estimation of nest density because nests likely vary intrinsically in their ability to be detected depending on nest stage. Nests in incubation likely have higher detection probabilities than those in the laying stage because flushing females provide the primary cue of nest occurrence during rope-dragging. Violation of this assumption has not been tested with nest data; however, evaluation of analogous capture-recapture models demonstrated that individual heterogeneity in capture probability may lead to a negative bias in abundance estimators (Kéry and Schaub 2012). We attempted to minimize the effects of this bias on inferences related to plot-level nest density and covariate effects by allocating nest searching efforts randomly across plots during the nesting period. This approach assumes any negative biases associated with individual heterogeneity in detection probability will be consistent across study plots.

The other assumptions of Dail and Madsen’s model, beyond those of the closed N-mixture model of Royle (2004) discussed above, are:

1. The number of nests at time t are dependent on the number at time t-1 (first-order Markov assumption)
2. The ‘survival’(ω) and ‘recruitment’ (γ) of nests at each plot are independent

Only the second assumption is relevant for the application of the DM models to nest density. As most passerines will renest after failed first nest attempts, γ is likely influenced by ω. Nevertheless, Dail and Madsen (2011) state that this assumption is somewhat relaxed in their model due to the autoregressive formulation of abundance where estimated abundance during a survey period is determined by the abundance during the preceding time period multiplied by both ω and γ. We have no way of formally testing this assumption with our data, but the assumption has the same potential implication for the standard application to avian point counts. Our primary objective for estimating ω and γ was to evaluate whether the population of nests was at equilibrium (e.g., closed) across survey periods; violations of this assumption are less of a concern than studies specifically interested in estimating and inferring patterns in ω and γ.
